# Expanding the Design Space for Fall Prevention in Acute Orthopedic Hospital Care: Human-Centered Design Study

**DOI:** 10.2196/73110

**Published:** 2025-10-02

**Authors:** Maria Ehn, Åsa Revenäs, Helena Tobiasson

**Affiliations:** 1 School of Innovation, Design and Technology Mälardalen University Västerås Sweden; 2 Ortopedic Clinic Västerås Västerås Hospital Region Västmanland Västerås Sweden; 3 Centre for Clinical Research Uppsala University Västerås Sweden; 4 School of Health Care and Social Welfare Mälardalen University Västerås Sweden; 5 School of Innovation, Design and Technology Mälardalen University Eskilstuna Sweden

**Keywords:** falls, prevention, hospital, technology, interaction

## Abstract

**Background:**

In-hospital fall prevention is a complex phenomenon most efficiently addressed via a wide range of multifactorial interventions. Technology may contribute, but research in this field has so far mainly focused on detecting falls. As a result, new knowledge from a system perspective is needed regarding when and how new technologies may support fall prevention among patients who have been hospitalized.

**Objective:**

This study aimed to explore and describe clinical practices in an acute orthopedic hospital ward for fall prevention from a system perspective; determine the needs and possibilities related to support for clinical practices for fall prevention; and test whether a framework for studying interactions between people, activities, contexts, and technologies can be used to support observations of complex phenomena such as clinical fall prevention.

**Methods:**

This qualitative study followed the principles of human-centered design while combining focused ethnography with a workshop. Eight health care professionals representing different staff categories in an acute hospital ward of an orthopedic clinic participated in on-site interviews or were observed in their clinical practice. Data from these events were subjected to qualitative content analysis to describe the clinical practices for fall prevention observed in terms of people, activities, context, and tools. In a workshop, a larger group of clinic personnel provided their views on fall prevention, described the activities and tools they observed to prevent falls, and discussed needs for further support.

**Results:**

This study determined that health personnel considered fall prevention in all their interactions with patients, which included a wide range of activities for fall prevention wherein staff categories played complementary roles. These staff-patient meetings were goal oriented, responsive, and patient centered. The staff often served as key “tools” in assessment, communication, and coaching, while digital tools (mainly computer-based software programs) were used for information retrieval, documentation, and communication. The personnel worked to prevent patient falls both during hospitalization and after discharge. They believed that the long-term perspective was much more difficult to address in their clinical practice, and they expressed a need for more homelike environments in the hospital.

**Conclusions:**

The view on technology-based in-hospital fall prevention can be broadened not only to mainly include monitoring and alarm systems, information systems in general, or computer-based information in particular systems but also to support activities performed by health personnel that engage patients in fall prevention. For example, tools such as these can be implemented in training involving daily activities and mobility within safe yet more homelike clinical contexts.

## Introduction

### Background

Falls are the most common adverse event reported in hospitals [1,2]. In-hospital falls may be effectively prevented by multifactorial interventions, but this may apply mostly to a subacute setting [3], and the interventions’ content can vary widely [4]. Home hazard minimization has been proven to reduce the number of falls among older patients recently discharged from hospitals, particularly if they had a history of recent falls [5]. However, interventions effective in preventing falls among older adults in general are not automatically transferable to this group [5]. So far, research on technology-based fall prevention [6] has mainly focused on systems for older adults living in the community [7] and resulted in few implementations in real-world situations. A systematic review of health technologies, which are defined as “...the application of organised knowledge and skills in the form of devices, medicines, vaccines, procedures and systems developed to solve a health problem and improve the quality of lives” [8], identified that highly heterogenous multicomponent or multifactorial interventions were most common in hospital settings, followed by technical devices to detect falls [9]. A few examples of fall detection technologies have been tested with patients [10-13], but most of them were emerging technologies that had not yet been tested on patients beforehand [9]. Nevertheless, an artificial intelligence–based motion alarm that notifies health personnel of fall risk situations has recently proved promising for in-hospital fall prevention [14]. To enable new complementary fall prevention technologies to evolve, a system perspective on fall prevention is needed.

### Prior Work

Previous studies on in-hospital fall prevention from a system perspective include the literature review by Taylor and Hignett [15], which analyzed risk factors and interventions to prevent in-hospital falls based on human factors and ergonomics. This review also presented a model describing the complex interplay between organizations, people, and environments related to in-hospital fall prevention [15]. The importance of human factors and ergonomics in the design of health care environments to support well-being and high performance has also been advocated by Hignett [16] and Hignett and Masud [17]. Through ethnographic observations and interviews of older persons on the subject of orthopedics in hospital wards, Mcvey et al [18] studied interactions between hospital staff and patients who have been hospitalized with cognitive impairments to engage patients in fall prevention. Mcvey et al [18] determined that patients who engaged with staff regarding fall prevention were able to express their needs and, to a certain extent, collaborate with the staff on fall prevention. The positive impact of patient and care team partnerships regarding fall prevention was also observed by Dykes et al [19] in their evaluation of the tailored prevention of inpatient falls tool. This tool, whose usability and patient centeredness have been improved in an iterative user-centered design process [20], clearly illustrates the importance of human factors in fall prevention. Similarly, Bianco et al [21] determined that insufficient dialogue with older adults about their personal goals results in the development of stigmatizing solutions for fall prevention that will not be accepted and implemented by the older users. Older adults’ experiences and perceptions of digital technologies for home-based fall prevention have also been investigated in a field study by Ogonowski et al [22]. This study concluded that the self-monitoring of results and self-assessment of fall risks made the system valuable and motivating for the users and that the successful integration of the system into daily life could increase the users’ quality of life, technical confidence, and awareness of measures for fall prevention [22].

The system perspective is also essential in human-centered design (HCD), which supports systems design based on human needs and functions from a holistic point of view [23]. HCD has been widely used in health research and innovations [24], such as in systems designed to prevent falls by assessing a person’s balance [25] and fall risk [26]. An essential part of HCD is developing activities to understand and specify the context of use, defined as “the combination of users, goals and tasks, resources and environment” [23]. Therefore, holistic studies of humans in their natural settings based on ethnographic principles are common in HCD [27]. Because ethnography is useful for producing detailed descriptions of phenomena, the method has also been suggested as suitable for health care research [28,29] and health care improvements [30]. Focused ethnography is a type of ethnography that studies specific aspects of field work such as interactions and activities; compared to classical ethnography, it typically involves shorter field visits, more extensive data and analysis work, and a greater focus on communicative activities [31]. This approach has been used to study older adults’ and their children’s perceptions of fall risks [32]. Workshops are another method commonly used in HCD for creating a common arena, enabling participants to discuss issues from different perspectives and areas of competence to develop a common experience within a complex phenomenon [33]. Workshops are widely used in health care innovation [34] and have been used by McKenzie et al [35] to support interprofessional learning to reduce the number of falls experienced by older adults. McKenzie et al [35] reported that their workshop participants believed the workshops provided them with the confidence to implement the fall-preventive practices presented and discussed in the workshops.

### Purpose of This Study

The three objectives of this study were as follows: (1) to explore and describe the clinical practices for fall prevention in an acute orthopedic hospital ward from a system perspective; (2) to determine the needs of patients and the possibilities of further supporting clinical practices for fall prevention; and (3) to test whether a framework for studying interactions between people, activities, contexts, and technologies (PACT) can be used to support observations of complex phenomena such as clinical fall prevention.

## Methods

The methods used in this study have been reported in accordance with the COREQ (Consolidated Criteria for Reporting Qualitative Studies) guidelines [36].

### Study Design

Because this study aimed to provide a detailed description of clinical practices for fall prevention, it had a qualitative research design based on HCD principles [23], signifying that it combined holistic studies of humans in their natural settings via focused ethnography [31] and a workshop [34].

### Theoretical Frameworks

#### PACT Framework

The PACT framework supports the analysis of people who use innovative technologies or tools, the activities that these people are willing to undertake, the contexts in which these activities take place, and the key features of meaningful innovative technologies [37]. PACT analyses have been used to transfer design knowledge from non–health care domains to health care to increase the usefulness of health care information systems [38].

#### Activities to Prevent Falls

Due to the well-documented need for effective strategies to prevent falls among inpatients during their high-risk hospital-to-home transition [39-41], this study included activities to prevent falls during the patients’ hospital stay and the hospitals’ efforts to prepare inpatients to take an active role in fall prevention after their discharge. On the basis of the clinical practices and guidelines recommended for Swedish caregivers and health care personnel [42,43], evidence-based methods to prevent in-hospital falls [44], and activities aiming to increase the patients’ possibilities to implement fall prevention actions at home [45] after their hospital discharge, this study has defined “activities to prevent falls” as the following:

- Fall risk assessments (related to fall histories, hospital personnel’s overall assessment, clinical instruments, and aspects related to the fall prevention activities mentioned subsequently)
- Fall prevention activities (related to medication, cognitive impairment, nutrition, care environment, physical exercise and mobilization, mobility aids, bathroom visits, and personal hygiene)
- Documentation regarding fall risks and fall prevention activities (from previous care providers regarding patients considered to be at risk, risk activities, risk moments, and any fall risk mentioned in a patient’s health record)
- Informational activities (regarding the fall risk of patients considered to be at risk as well as handover)
- Activities supporting patients’ physical activity and home modifications to prevent falls after hospital discharge

### Ethical Considerations

On the basis of the Swedish Act Concerning the Ethical Review of Research Involving Humans, the research in this study (ethnography and workshop) did not require ethical vetting. An application for the ethical review of the ethnographic work was submitted to the Swedish Ethical Review Authority. The written decision from the authority (Dnr 2022-05412-01) confirmed that this research did not require ethical vetting, and the authority stated that it did not see any ethical difficulties related to the research. All the research performed in this study was conducted in accordance with good research practices. The persons interested in participating in the clinical observations and on-site interviews received oral and written information about the study, and informed written consent was collected from all participants before their participation. The workshop participants received oral and written information through a Microsoft PowerPoint presentation before completing the questionnaire and provided written consent on the questionnaire to participate in the research.

### Research Setting

This study was performed in an orthopedic clinic at a hospital located in a medium-sized Swedish city with approximately 150,000 inhabitants. The clinic performed pre and postoperative rehabilitation and outpatient and inpatient care, including elective and acute surgical operations. The ethnography was conducted at the clinic’s acute hospital ward, and the user workshop was held during a seminar open to staff from the entire clinic. The study was performed by ME, a researcher in medical and health technology with expertise in user-centered development. ÅR, a researcher in physiotherapy also working at the orthopedic clinic, and HT, a human-computer interaction researcher, participated in peer debriefing. Both had clinical experience as physiotherapists. All 3 researchers are female.

### Participants and Recruitment

#### Participants in Clinical Observations and On-Site Interviews

The 8 participants came from the approximately 100 health care workers in the acute ward (Table 1). They had been recruited according to principles of purposive sampling, combining criterion-based (personnel active in fall prevention within a specific clinical setting) and stratified purposeful (personnel representing a variety of staff categories) sampling strategies [46]. The participants represented the following staff categories: assistant nurse, clinical pharmacist, nurse, occupational therapist, physician, and physiotherapist. One of the nurses was a discharge coordinator. The study aimed to recruit at least 1 person per staff category and cover both morning and afternoon shifts.

**Table 1:** Data collection events and participants.

Events specified by consecutive number		Participants	Time
**Clinical observations**			
	1	Physiotherapist and occupational therapist	Morning
	2	Assistant nurse	Afternoon
	3	Nurse	Afternoon
	4	Nurse	Morning
	5	Physician	Morning
**On-site interviews**			
	6	Clinical pharmacist	Morning
	7	Discharge coordinator	Morning
**Workshop**			
	8	Representatives of several health care occupations (refer to the Workshop Participants subsection)	Lunch

To recruit participants, ME presented the study at staff meetings and on the clinic’s intranet. Thereafter, she booked appointments with 5 interested staff members by email and found 3 interested staff members while visiting the ward to see if any health personnel would volunteer to be observed.

#### Workshop Participants

The participants were clinical staff members who attended a research seminar at the orthopedic clinic and filled in a questionnaire. The participants represented the following health occupations: assistant nurse, health counselor, medical secretary, nurse, occupational therapist, physician, and physical therapist.

### Data Collection and Curation

#### Overview

An overview of the data collection events and their respective participants is presented in Table 1. The methods used in each event are thoroughly described subsequently. The clinical observations and on-site interviews were performed from February to May 2023, and the workshop was held in November 2023.

#### On-Site Observations and Interviews

On-site observations of health personnel were performed on 5 different occasions (Table 1). Each time, a researcher followed 1 or 2 participants for 2 to 4 hours to observe them during routine patient care. Apart from the participants’ health care occupation, no personal information was collected from any of the persons observed. Instead, general types of attributes relevant to the persons’ involvement in fall prevention activities were observed along with any attributes the health care personnel discussed and considered in their work with patients. The observations were documented via written notes in a structured protocol that recorded the activities observed to prevent falls, any other persons involved, and the context in which the activities took place (Multimedia Appendix 1). At the end of each observation, the researcher asked the participant a few questions regarding whether something unusual had happened or the person had done anything more or less than usual during the shift; whether the person had used any specific templates or forms to provide or receive a report before, during, or after the shift; and whether the person would like to correct or add something to the researchers’ observational notes by following a brief interview guide (Multimedia Appendix 1). Of the 8 health care personnel, 2 (25 %) who worked in an office participated in unstructured on-site interviews for 60 to 90 minutes instead of the observations (Table 1). These interviews focused on the same aspects as the observations. All the interviews were documented through written notes. Audio recordings were avoided to help participants feel more at ease. Before the first observation, the researcher examined an empty patient room and used the observation protocol to document what they saw via photos and written notes. After each observation (the same or following day), the researcher wrote down their reflections, ideas, and questions in notes, which were later transferred to the observation protocol. Thereafter, the researcher prepared a text integrating the observational data and their own reflections (based on the protocol), the interview data, and any additional information that they recalled during the writing process.

#### Workshop

Data were collected during a research seminar for the entire orthopedic clinic’s staff. The seminar included presentations of 3 projects, of which 1 was the ethnographic work from this study The presentation of our ethnographic work provided a brief background on in-hospital fall prevention and current research regarding supporting technologies; the study’s approach, aim, expected results, and methodology; how the researcher defined the “activities used to prevent falls” in the study; the potential benefits of staff participating in the study; and the clinical observations. Next, the researcher presented the preliminary results regarding the observed activities that the respective health professions implemented to prevent falls, as well as the tools used during these activities. Finally, the researcher introduced the workshop approach, which included group discussions on the 3 questions related to the staff’s views on the definition of “activities to prevent falls” and how these activities contribute to preventing falls both in the short and long term; whether adjustments were needed in the presentation of in-hospital activities to prevent falls as well as the tools supporting the activities; and what may be needed to further develop any aspect of the activities, context, or tools to further improve the work to prevent falls (Multimedia Appendix 2). Participants were also invited to contribute their own responses to the research by providing written consent and submitting the completed forms to the researcher.

### Data Analysis

#### On-Site Observations and Interviews

A qualitative content analysis was performed based on principles described by Elo and Kyngas [47] using Microsoft Excel as the software tool for the categorizations. All descriptive texts integrating the observational and interview data, as well as the researcher’s own reflections for each individual observation and on-site interview, were first analyzed separately in an unconstrained categorization matrix based on PACT [37]. In this adaptation, the category “tools” was used in place of “technology,” as originally defined in the PACT model. ME, who was familiar with the material from data collection and curation, read the running text and coded phrases based on their content as people, activities, contexts, or tools. Data categorized as people and context were further categorized into health personnel; patients; external people; and organizational, social, and physical environment, respectively. Data from the people and context subcategories, as well as the tools and activities categories, were analyzed following principles of inductive content analysis [47]. Thereafter, the main and subcharacteristics of the matrix elements related to people, contexts, and tools from all observations and interviews, as well as from the activities of all health care occupations, were reviewed and integrated by ME, who subsequently wrote new descriptive texts for all characteristics in the individual matrix elements. ÅR and HT, who had not been involved in data collection or curation, participated in peer debriefing, during which they gave their views on the descriptive texts, coding, and categorizations, as well as the overall analysis process [32,48]. The activities from each health care personnel category were visualized, and information on the type of tool used in specific activities was added to the visualizations. In some cases, the titles and structures of the categorization’s main and subgroups were modified during the writing process.

#### Workshop

Free-text answers from the groups on the open-ended questionnaire were summarized in a table. The answers were read and discussed by all the authors.

### Strategies Used in the Study to Achieve Trustworthiness

#### Overview

The strategies this study applied to achieve trustworthiness are described subsequently and based on the parallel criteria for trustworthiness in qualitative research developed by Lincoln and Guba [49], who recommend applying strategies to qualitative research that better achieve their 4 criteria for trustworthiness (credibility, transferability, dependability, and confirmability). Application of the 4 criteria by Lincoln and Guba [49] in qualitative research has been reported by other researchers, for example, by Enworo [50] and Forero et al [51].

#### Credibility

This study applied the following strategies to achieve credibility: *prolonged and varied engagement with the setting*, by ME visiting the clinical environment on several occasions over several months during different activities; *in-method triangulation*, by integrating observations, interviews, and the workshop; *data source triangulation*, by involving personnel representing different staff categories within different settings at the orthopedic clinic; *peer review*, with ÅR and HT reviewing data, analysis process, and the result; and *member check*, which was performed through the brief interviews at the end of the observations and the workshop, during which the study findings were presented and discussed with a broader group of health personnel.

#### Dependability

To achieve dependability, this study applied the following 2 strategies: *rich description of study methods*, as reflected in the methods description guided by the COREQ checklist [36], and *member check* (refer to the description mentioned earlier under the Credibility subsection).

#### Confirmability

To achieve confirmability, this study applied 2 strategies: *reflexivity*, via ME’s self-reflections after each observation and on-site interview, the researchers’ common reflections in the peer review meetings, and the workshop participants’ reflections on the study results presented; and *triangulation* (refer to the description mentioned earlier under the Credibility subsection).

#### Transferability

To achieve transferability, this study applied the 2 strategies: *purposeful sampling*, by combining criterion-based (personal active in fall prevention in a specific clinical setting) and stratified purposeful (personal representing a variety of staff categories) sampling strategies, and *thick description*, in which the integration of data collected from several sources (observations, interviews, reflections, and the workshop) on several different occasions contributed to a rich and detailed description of in-hospital fall prevention.

## Results

### Clinical Observations and On-Site Interviews

#### People

The people involved in activities to prevent falls were health personnel, patients, and external persons. Main and subcategories are presented in Multimedia Appendix 3. The clinical practices implemented by individual members of the *health personnel* depended on which health occupation they were licensed in, their amount of clinical experience, whether they held specific positions in management or coordination, and their individual competencies. Moreover, the health personnel customized fall prevention activities for the individual *patients*, who were all adults of varied age. The individualization was based on the patient’s overall health status, which could include both the reason that the patient had sought acute orthopedic care and any other potential additional health problems. The patient’s personal factors, such as communication, life situation, self-awareness, and preferences, also needed to be taken into account. In some cases, the patients and health personnel also interacted with *external persons* in fall prevention. This could include health personnel from other clinical units in patient transportation, medical consultations, and referrals to primary care, as well as municipal staff in planning continued care along with the patients’ family members.

#### Activities and Tools

**Figure 1:**
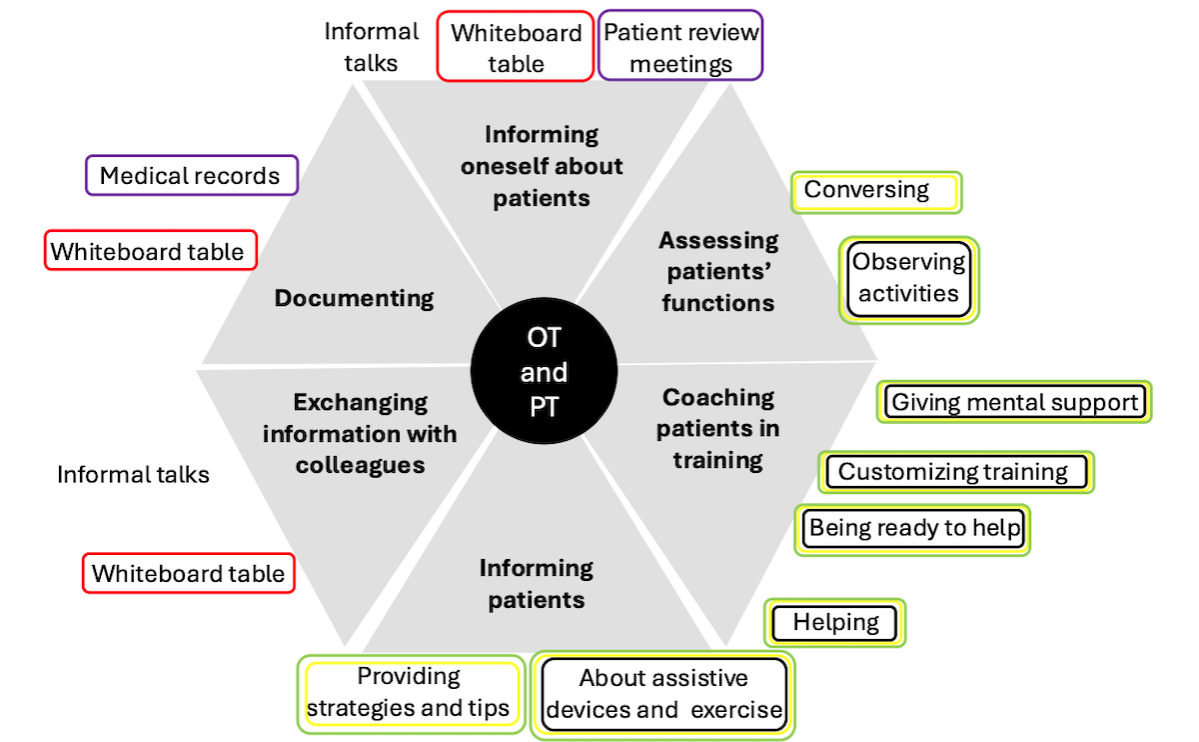
The clinical practices for fall prevention performed by physical and occupational therapists, with the type of activity (main categories) presented in bold text in gray fields. Specific activities (associated subcategories) are presented in normal font text outside their respective gray field. The supporting tools are represented by circles around the activities. The color of the circle indicates the type of tool used: purple represents computer-based systems, red represents physical tools and systems, green represents the staff, black represents mechanical aids and assistive devices, and yellow represents physical barriers and mechanical tools. OT: occupational therapist; PT: physical therapist.

**Figure 2:**
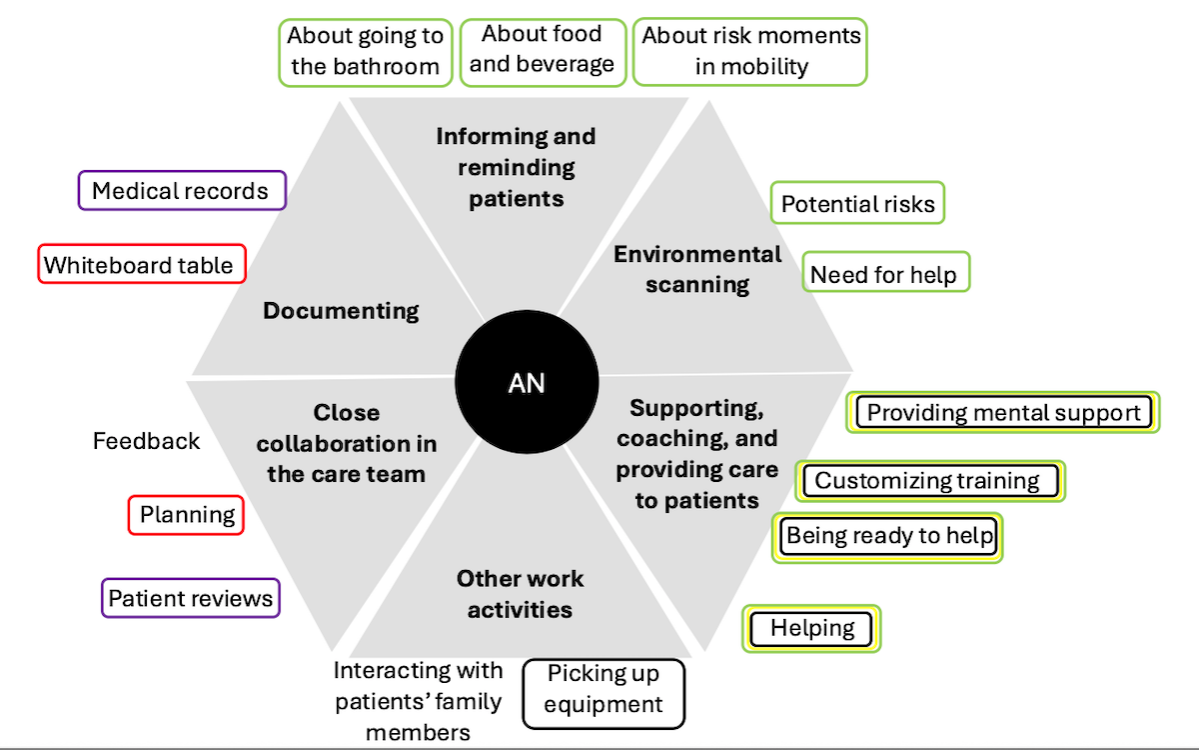
The clinical practices for fall prevention performed by assistant nurses, with the type of activity (main categories) presented by bold text in gray fields. Specific activities (associated subcategories) are presented in normal font text outside the respective gray field. The supporting tools are represented by circles around the activities. The color of the circle indicates the type of tool used: purple represents computer-based systems, red represents physical tools and systems, green represents the staff, black represents mechanical aids and assistive devices, and yellow represents physical barriers and mechanical tools. AN: assistant nurse.

**Figure 3:**
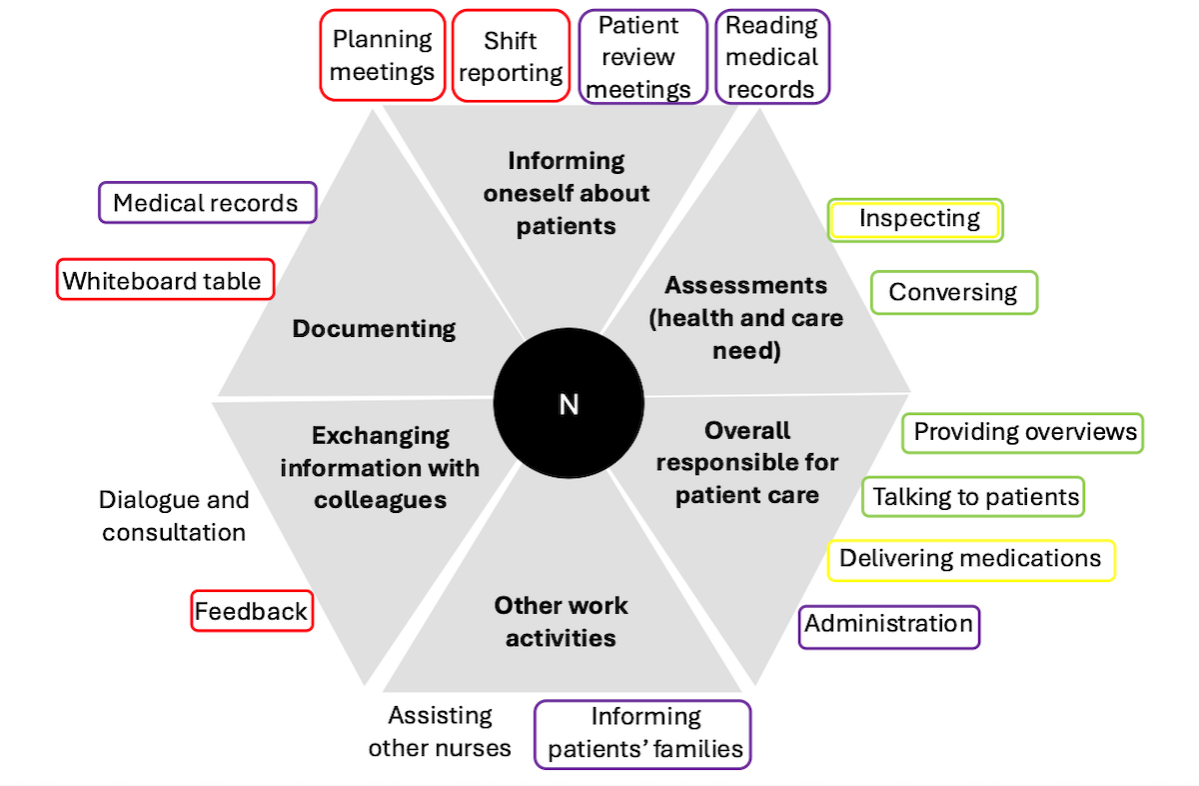
The clinical practices for fall prevention performed by nurses, with type of activity (main categories) presented by bold text in gray fields. Specific activities (associated subcategories) are presented in normal font text outside the respective gray field. The supporting tools are represented by circles around the activities. The color of the circle indicates the type of tool used: purple represents computer-based systems, red represents physical tools and systems, green represents the staff, black represents mechanical aids and assistive devices, and yellow represents physical barriers and mechanical tools. N: nurse.

**Figure 4:**
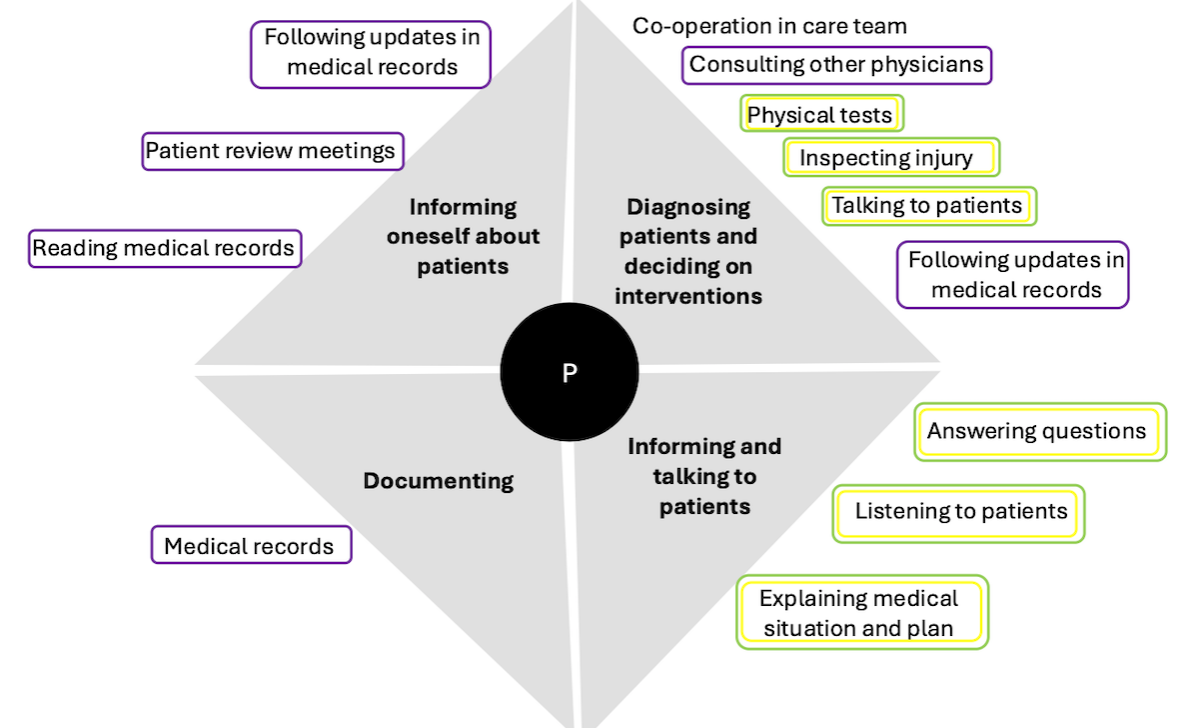
The clinical practices for fall prevention performed by physicians, with the type of activity (main categories) presented by bold text in gray fields. Specific activities (associated subcategories) are presented in normal font text outside the respective gray field. The supporting tools are represented by circles around the activities. The color of the circle indicates the type of tool used: purple represents computer-based systems, red represents physical tools and systems, green represents the staff, black represents mechanical aids and assistive devices, and yellow represents physical barriers and mechanical tools. P: physician.

**Figure 5:**
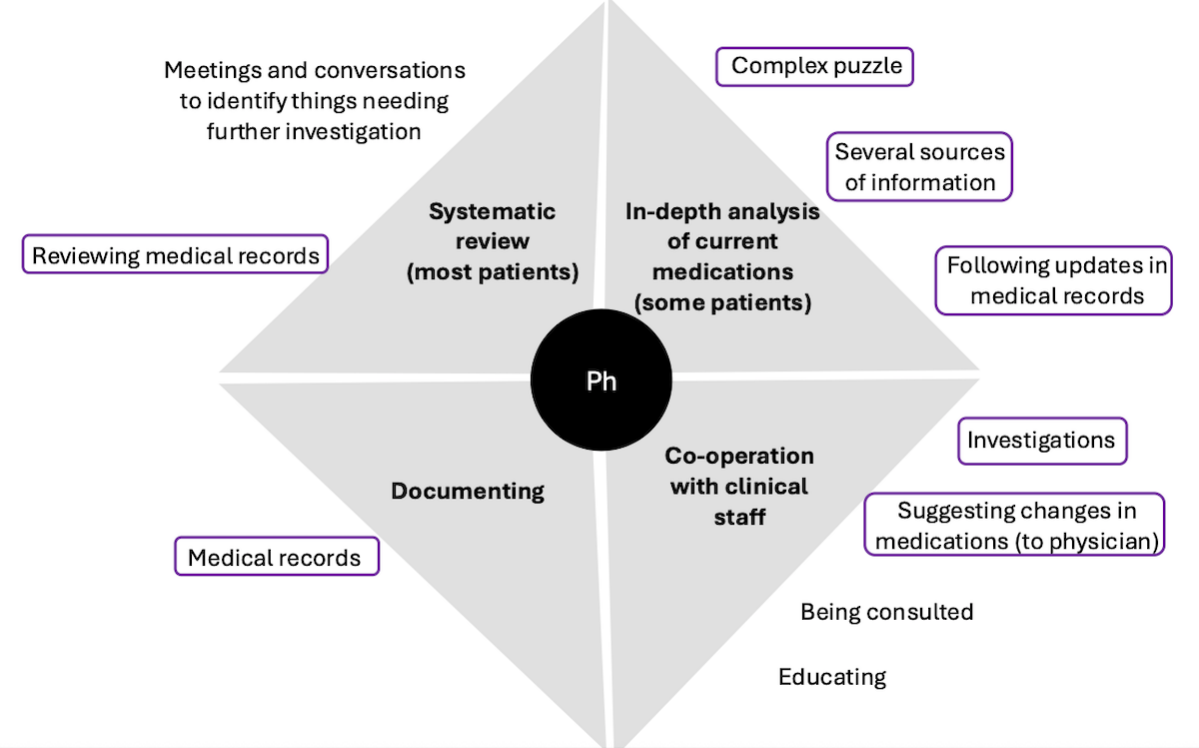
The clinical practices for fall prevention performed by the clinical pharmacist, with type of activities (main categories) presented by bold text in gray fields. Specific activities (associated subcategories) are presented in normal font text outside the respective gray field. The supporting tools are represented by circles around the activities. The color of the circle indicates the type of tool used: purple represents computer-based systems. Ph: clinical pharmacist.

**Figure 6:**
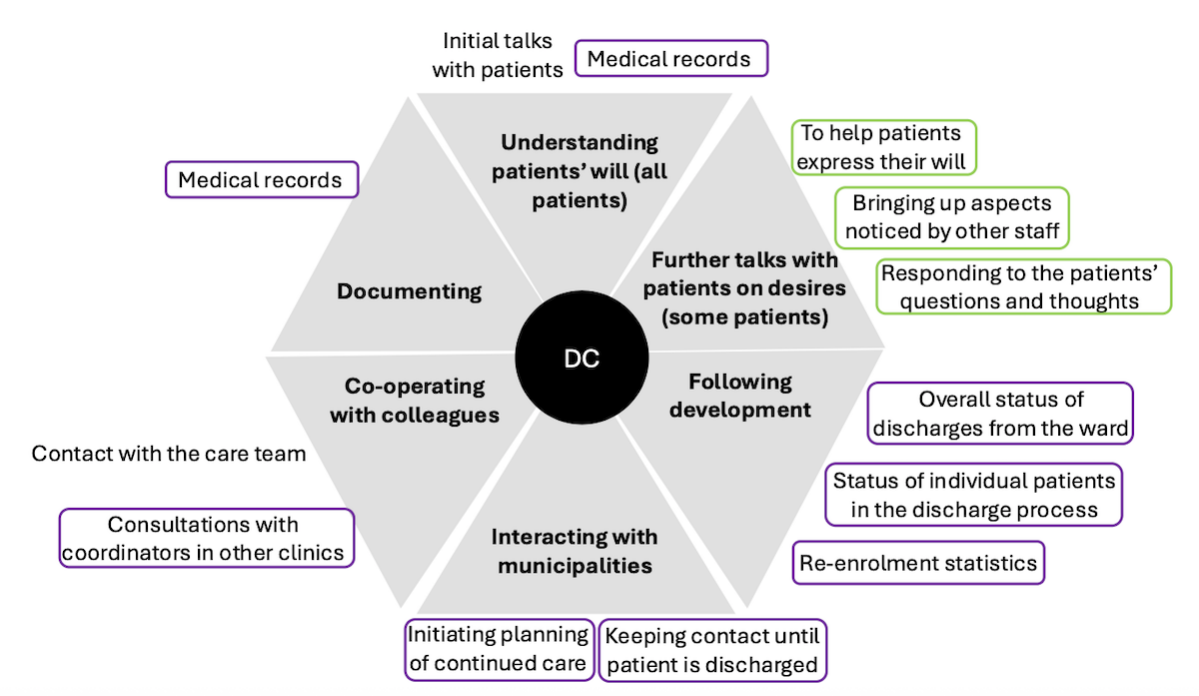
The clinical practices for fall prevention performed by discharge coordinators, with type of activities (main categories) presented by bold text in gray fields. Specific activities (associated subcategories) are presented in normal font text outside the respective gray field. The supporting tools are represented by circles around the activities. The color of the circle indicates the type of tool used: purple represents computer-based systems and green represents the staff. DC: discharge coordinator.

Fall prevention activities and supporting tools are presented by staff category in Figures 1-6 and in the descriptive texts subsequently.

The physical and occupational therapists mainly supported patients in fall prevention during mobility training and activities in daily life (ADLs; Figure 1).

The therapists prioritized meeting patients who were new or close to being discharged. Their meetings focused on evaluating and supporting the patients as they underwent training in tasks essential for ADL. To initiate this, the therapists asked the patient to perform basic tasks, such as sitting up in bed. As the patient accomplished tasks, the therapists gradually introduced the patient to more challenging tasks, such as rising, standing, walking with mobility aids, and performing ADLs. Although the therapists encouraged the patients to try these tasks independently, they were constantly ready to support them immediately if needed. The therapists assessed the patient’s functions and well-being by observing how the patient seemed to feel (eg, if they were affected by pain) and by listening to what the patient said (eg, regarding well-being, pain, mobility, their living situation, and assistive devices). They provided the patient with coaching and mental support, calming and encouraging them, based on the patient’s individual needs. As they customized the training to the patient’s current abilities, they prevented the patient from experiencing “failures.” Tasks that seemed too challenging in training on 1 occasion could be postponed until an up-coming meeting or be supported by assistant nurses later the same day. The therapists gave advice related to safe mobility habits, ADL, and body positioning; provided practical help to patients; and eliminated safety risks in the physical environment near the patient.

Assistant nurses provided close care and support to patients, such as when they got dressed, visited the bathroom, and engaged in mobility training (Figure 2).

The assistant nurses moved along the corridor, scanning for patients in need of support, such as helping them go to the bathroom, and performing scheduled visits to see patients and to take food orders. They assisted patients with food orders by conversing, checking their documentation, and ensuring access to beverages. They also scanned for potential safety risks in the patient rooms and modified their physical environment by removing tripping hazards; helping patients to vary body position when needed; and reminding the patients about food, beverages, and bathroom visits, etc. They also supported patients in mobility training by reminding them, for example, of how to safely position their feet or lock their walker’s wheels. While interacting with the patients, the assistant nurses were attentive, responsive, encouraging, calm, and clear and always one step ahead of the patient while focusing on safety. The assistant nurses collaborated closely with nurses, assisted colleagues, and interacted with the patients’ family members at the ward.

Nurses were responsible for the care of a small group of patients, and there were approximately 4 to 6 patients per nurse. Their work included planning follow-up care, which in many cases involved prevention (Figure 3).

The nurses consistently kept themselves well informed of their patients’ history and status through the patients’ medical records, their own written notes from previous shifts, conversations with staff members from the outgoing and current shifts, and talking to patients during care activities and patient review meetings. Moreover, they continuously informed the care team’s staff and interacted with the care team, physicians, and care coordinators through discussions, consultations, and information exchange. Together with the care team, they planned their activities and documented them on a whiteboard table and in their own written notes. Their interactions with assistant nurses, who carried out most of the point-of-care activities, were crucial and included continuous 2-way reporting performed orally and on the whiteboard table. The nurses documented all care activities provided in the patients’ medical records, including notes from patient review meetings when necessary. They performed structured patient rounds to introduce themselves, check the patients’ health status, and deliver medications and blood transfusions to patients. They also administered and followed up on medical and care activities, assisted colleagues, and talked to the patients’ family members.

Physicians were responsible for the patients’ medical care, including providing medication that could be closely related to fall prevention (Figure 4).

The ward physician was available in the ward during the daytime. They had support from a more senior colleague who, after having attended patient review meetings with patient care teams and participated in the clinical rounds at the ward, was accessible over phone. The ward physician consistently stayed well informed by reading medical records, leading the patient review meetings, and interacting with all the patients during their clinical rounds. In the patient review meetings, the care team informed the physicians about events from the past shift. In some cases, the ward physician reported on consultations with specialists in related domains. On the basis of reports, discussions, and consultations, the senior physician decided on the medical interventions, which the ward physician documented in the medical records. Thereafter, the physicians met all the patients in their rooms during their daily clinical rounds. Here, they assessed the patients’ state of well-being and cognition based on conversations with them and their muscular and neural functions based on physical examinations, which included observations of the patients’ responses to specific stimuli, visual inspections of any bandages, and subcutaneous bleeding. The specialist physician informed the patients of the results of their examinations, their medications, and any plans for ongoing evaluations and interventions. The physicians demonstrated where injuries were located by pointing at their own body. Before their discharge, patients received a written summary of the examinations and interventions they underwent, the results, and the plan for continued care. The physicians listened to the patients’ experiences, answered questions, and asked them if they felt comfortable with the plans. In their conversations with patients, the physicians appeared clear and calm, and the patients showed them confidence and respect.

The clinical pharmacist supported the physicians and other health personnel by answering questions related to pharmacology, including those related to fall risks (Figure 5).

The pharmacist systematically reviewed the drugs prescribed for most patients to identify any need to change them based on the information from the medical records, a national system for prescribed medications, patient review meetings with physicians and the patient care team, and consultations with the discharge coordinators. They screened for potential drug-related medical issues by checking the patients’ medication lists and physiological parameters in the medical records. They also made thorough reviews of drug prescriptions for patients at risk of having drug-related health problems, such as patients with polypharmacy issues. These reviews were complex puzzles balancing combinations of risks and benefits. They focused on medications with documented issues affecting fall risks, either by themselves or in combination with other drugs, particularly among older patients. This may include prescribed combinations of medications that may cause drowsiness and fatigue, drug prescriptions deviating from general recommendations or those lacking evaluation, prescribed doses of antihypertensive drugs high enough to cause dizziness, and diuretics prescribed to be taken at certain times that could increase the risk of nocturnal bathroom visits. The analysis was done by manually comparing complex information from separate systems on the computer screen. The pharmacist documented suggested changes in drug prescriptions in the patients’ medical records for the physicians to consider. The physicians then decided whether and at what point a change should be implemented. Changes more suitable to implement after the patients’ hospital discharge were referred to physicians in primary care.

The discharge coordinators supported the patients in their discharge and safe transition home by addressing fall risks and considering the patients’ desires (Figure 6).

The discharge coordinators gathered information on the patients’ desires by reading their medical records and talking to them. Some patients were able to clearly describe their living and occupational situations and express their requirements for discharge in a single conversation. Others needed more time to process what type of help they wanted or needed after their discharge. Therefore, the coordinator talked to them again to guide them in their decision-making process by asking them concrete questions about their daily life (eg, home environment, ADL capacity, etc) and by describing the possibilities of home-based help for patients interested in such support. Because the coordinators had a professional role different from that of the care team, they could spend more time talking to the patients about things they brought up or about needs that other staff members had noticed. The coordinators cooperated closely with the care team staff, listened to their assessments, and reminded them to carefully document the patients’ state of well-being and activities in their health records. They also cooperated with municipal staff to plan for the continued care of patients who had given their consent and patients who needed such care but were unable to give their consent. The coordinators conveyed the patients’ desires via telephone and a computerized system to the municipal care administrators, who decided on which activities the patient were entitled to. The coordinators also communicated with the patients’ family members, who sometimes contacted the clinic to emphasize their relative’s needs and were then reminded to talk to their relative about his or her desires. Although the coordinators tracked 30-day readmission statistics, their involvement ended once the patient was discharged.

The tools used in the wide range of activities described earlier to prevent falls (Figures 1-6) can be categorized into the 5 main categories:

- Computer-based systems, for example, medical records software (purple in figures)
- Physical tools and systems, for example, the care teams’ whiteboard table (red)
- The staff, for example, their sight and hearing while assessing patients (green)
- Mechanical aids and assistive devices, for example, walkers for mobility (black)
- Physical barriers and mechanical tools, for example, sliding mats when lifting (yellow)

These are all summarized in Multimedia Appendix 4.

#### Context

**Figure 7:**
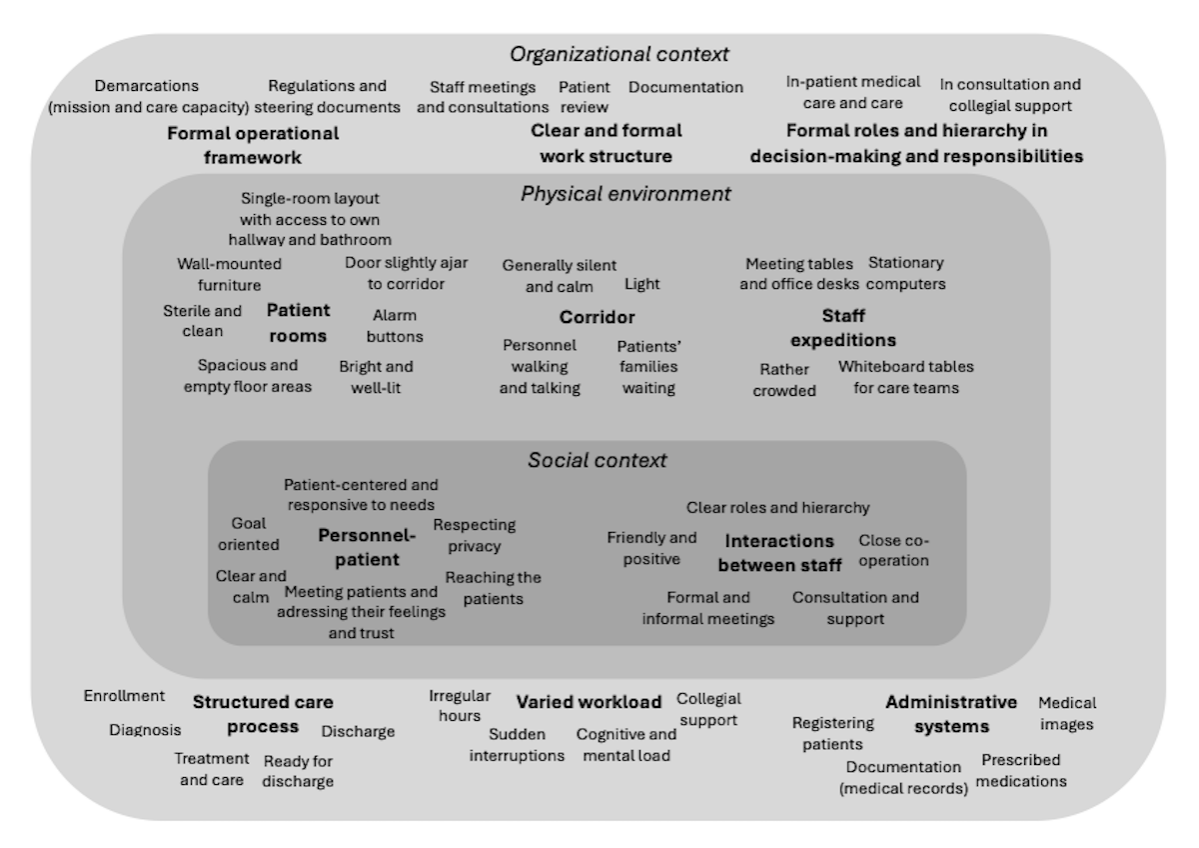
A description of the context, in which the main categories (in bold) and associated subcategories (nonbold) are summarized for the organizational context (outer circle area), physical environment (middle circle area), and social context (inner circle area).

Within the PACT framework, fall prevention is described in terms of the physical environment as well as the social and organizational contexts. The main and subcategories are presented in Multimedia Appendix 5 and Figure 7.

The organizational context of fall prevention was highly structured in the work itself and the patient care process, and it was formal in the operational framework and the health personnel’s roles and responsibilities. Other essential elements within the organizational context included a varied workload, which could be increased by mental and cognitive loads, sudden interruptions, and irregular work hours. The workload could also be reduced through collegial support and several administrative computer-based systems that the personnel used regularly.

The physical environment for fall prevention included the location where the health personnel met patients, which was primarily the patients’ rooms; the location where the patient care teams met, which was primarily the office where the physicians did administrative work and held teaching meetings with the care team staff; and the office where nurses, licensed practical nurses, as well as physiotherapists and occupational therapists did administrative work, planning, and reporting. Both offices had meeting tables and office desks with desktop computers, but the physicians’ office had a wall-mounted screen to project the physician’s computer display during the teaching meetings. The care team’s office had a large whiteboard on which the status and planned activities for all patients were documented. The location dedicated to the care teams (which included the patient rooms, day room, and health personnel team offices) was connected to long corridors, which were brightly lit, mostly silent, and calm. In the corridor, the health personnel could be seen walking on their way to see a patient or a colleague, occasionally pausing to have a quick word with a colleague. Occasionally, a patient walked along the corridor, and sometimes, the patients’ families waited in the corridor outside a patient’s room. Patient rooms were on 1 side of the corridor, with most of the doors opened slightly. Most patient rooms were single rooms with a small hallway leading into a spacious and bright bedroom with double windows and large open spaces. The walls and ceiling were painted white. The floor was light gray and free from raised sills and thresholds. The patients’ rooms were well lit by several light sources on the ceiling and on the wall over a height-adjustable bed, which could be raised or lowered via a panel on 1 side. The bed also had height-adjustable safety rails on the sides. A wired patient alarm control unit was placed beside the bed, mostly near the patient on a mobile bedside table whose tabletop could be placed over the bed. The bedrooms also contained a basin along with disinfectants, a chair, and a large television, all of which were wall mounted. All the patient rooms had their own spacious bathrooms with a shower area, and they were equipped with several supportive grips placed at strategic places for safely sitting down and rising from the toilet seat and moving to and standing in the shower. Alarm buttons were placed near the toilet seat, door entrance, etc. A movable laundry basket was the only item set on the floor.

The social context for fall prevention included settings for the health personnel’s interactions with patients, colleagues, and outside persons. The health personnel interacted with patients in different constellations, mostly when 1 or 2 members of the health personnel visited the patient’s room. The patient-staff interactions were patient centered, goal oriented, and empathetic. In sensitive situations, the personnel closed the door to the corridor to protect the patient’s privacy. To reach out to the patients, the personnel needed to be perceptive, flexible, and prepared to handle difficult topics brought up by patients in a professional way. The personnel also had to act in a calm, clear, direct, and pedagogic manner to enable the patients to understand and assimilate information, as well as to instill trust and confidence. The patients listened to the personnel and occasionally expressed their feelings toward them, including trust and confidence. Interactions between health personnel took place between colleagues in the patient care team or between the patient care team and staff in specific positions. Here, their roles and responsibilities were noticeably clear. The health personnel consulted each other in both formal and informal meetings within a friendly and positive atmosphere. On the basis of their distinct roles, the health personnel could also complement each other when in contact with a patient. The personnel’s interactions with external persons were also patient centered. The personnel stated that they sometimes needed to remind the patients’ family members that care planning must be based on the patient’s own preferences. Moreover, they stated that they had to respect and confide in the division of responsibilities between the clinic and municipality.

### The Staff’s View on Fall Prevention, Descriptions of Observed Activities and Tools, and the Need to Further Support Fall Prevention

A larger number of staff members from the orthopedic clinic provided their views on the analysis of observed activities to prevent falls and the tools used to support these activities, which were presented in the Activities and Tools subsection earlier. The staff provided feedback in groups representing specific health staff categories, which included assistant nurse, health counselor, medical secretary, nurse, occupational therapist, physician, and physical therapist (Table 2). As can be seen in Table 2, the staff supported the broad definition of activities to prevent falls and said that they considered fall prevention in all interactions with patients. They also stated that long-term fall prevention was more difficult due to short hospital visits and their lack of influence over the home environment. They agreed on the presentation of activities to prevent falls and the tools used. The staff described the need to further support fall prevention, such as by using more practical “real-life tools” and “making the ward’s physical environment more similar to the home environment.”

**Table 2:** Health personnel’s views on fall prevention, descriptions of observed activities and tools used to prevent falls, and identified needs for further support.

Group	View on the definition of “activities to prevent falls” as activities contributing to preventing falls both in the short and long term	View on whether adjustments are needed in the presentation of in-hospital activities to prevent falls and the tools supporting these activities	View on the need to further develop any part of the activities, context, or tools to further improve the work to prevent falls
Group 1 (occupational therapist, physiotherapist, and physician)	“It is possible to prevent falls in the short term at the hospital but more difficult to prevent falls in the long term because hospital stays are short and we cannot control the home environment.”	“No.”	“Make the hospital environment more like the home environment by incorporating features such as doorsteps, carpets, narrow spaces, and doors without automatic opening mechanisms.”
Group 2 (assistant nurse and nurse)	“We agree.”	“No.”	“No.”
Group 3 (medical secretary and nurse)	“Good, important work”	“Feels like you got it all.”	Continue to develop more practical, real-life tools and access to the latest news
Group 4 (health counselor, physiotherapist, and occupational therapist)	“We consider fall prevention in all interactions with patients at the ward and automatically assess fall risks in everything we do together with the patients.”	“No.”	—^a^

^a^Not available.

## Discussion

### Principal Findings

**Figure 8:**
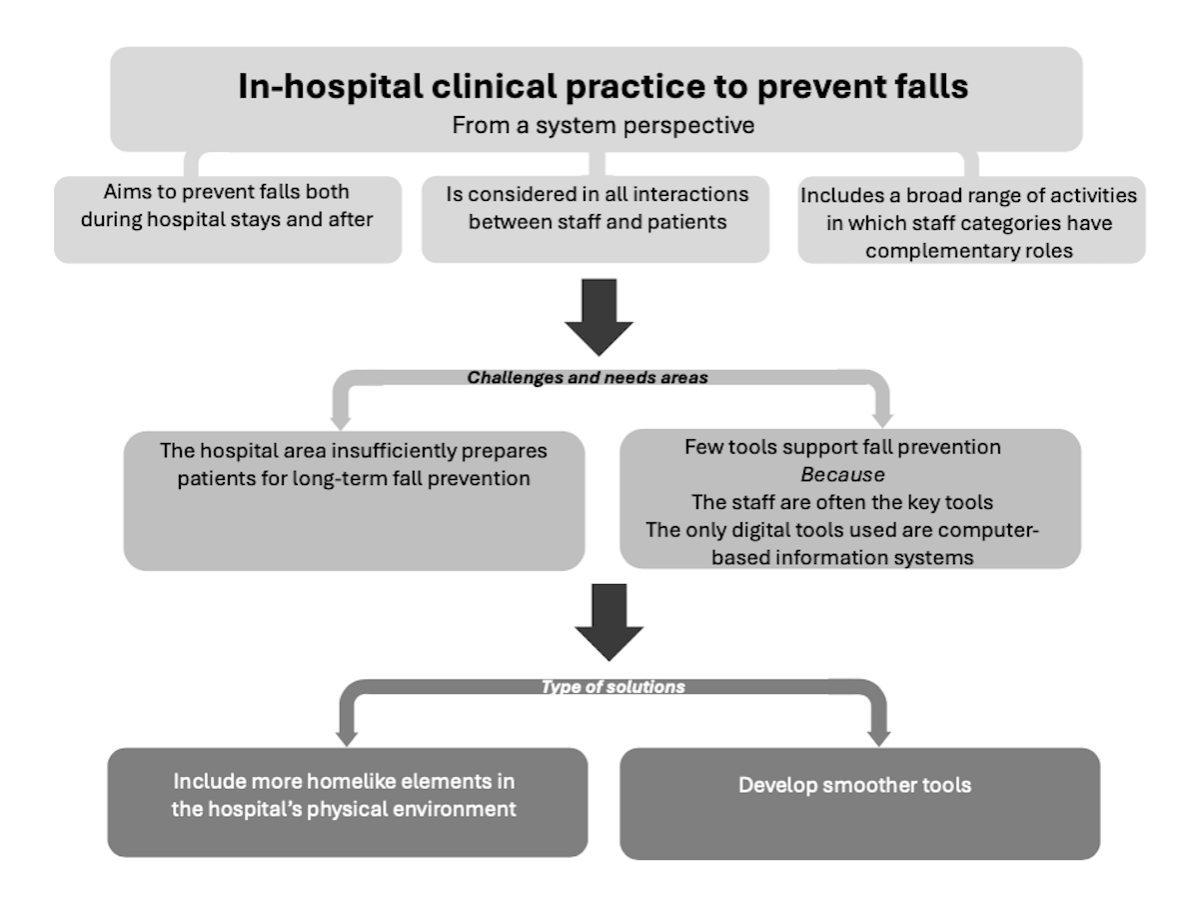
A summary of the main findings of the study, including key aspects of the in-hospital clinical practice to prevent falls studied from a system perspective based on the people, activities, contexts, and technologies framework, along with areas identified for further support and the types of solutions required by staff.

The principal findings of this study (summarized in Figure 8) showed that the health staff had a holistic view of fall prevention, which was recognized in the clinical observations of the people, activities, context, and tools in a clinical ward. This view was also expressed verbally by the personnel in a workshop. As a result, the study determined that the PACT framework can be useful to support the observation of complex phenomena such as clinical fall prevention. The health personnel stated that they automatically assessed fall risks and considered fall prevention in all their interactions and activities with patients to prevent falls both in the hospital setting and after hospital discharge. It was clearly seen in the observations that the health personnel strived to make the hospital stay safe for the patients and to prepare the patients for a safe transition home (or to a care home). This was observed in the wide range of activities in which the respective health staff categories had complementary roles. For example, physiotherapists and occupational therapists focused on mobility training and ADLs in their assessments and training with patients, while assistant nurses screened the patient environments for risk factors, supported patients in mobility training, and cooperated with nurses to provide all the patients with effective and safe care. The pharmacist and the physicians reviewed medications prescribed to each patient with the aim of minimizing fall risks, while the discharge coordinators interacted with patients to plan a safe home transition. In the workshop, the health personnel mentioned that the short hospital stays and their own lack of control over the patient’s home environment made it more difficult for them to address long-term fall prevention in their clinical practice; therefore, they saw a need for making the hospital environment more homelike by incorporating elements such as doorsteps, carpets, narrow spaces, and manually operated doors. Observations in the wards confirmed that the physical environment was clean, spacious, and free from homelike physical elements that could both increase fall risk as well as enable ADL and mobility training in situations representative of the home setting. The responsive, goal-oriented, and patient-centered interactions between the patients and personnel, which seemed very important for engaging the patients in fall prevention, were also observed in the ward. The observations revealed that although the personnel used different tools in their work and possessed a broad range of skills and functions, they often served as key “tools” themselves in many of their fall prevention interactions by assessing and communicating with and coaching the patients, all while using digital tools (mainly computer-based software) for information retrieval, documentation, and communication. The personnel expressed that they were interested in more practical, real-life tools for fall prevention.

### Limitations

This study has several limitations. The method used (ie, collecting clinical practice data relative to fall prevention via observations, interviews, and the workshop without collecting any personal data from the participants except for the health personnel’s staff category) limited the results because they encompassed only observable actions, verbal expressions, and contexts related to the people, activities, and tools involved in fall prevention. Consequently, no conclusions were drawn in this study regarding the staff’s personal experiences; feelings; mental models; or the psychological, social, and attitudinal attributes related to fall prevention. Moreover, the study explored the health staff’s perspectives on fall prevention, and no data were collected regarding the patients’ perspective other than what the patients expressed and did in their interactions with the staff.

Although the study applied several strategies (described in the Methods section) to achieve trustworthiness, the study also had methodological limitations. For example, the sampling, performed via criterion-based (personnel involved in fall prevention within a specific clinical setting) and stratified purposeful (personnel representing a variety of staff categories) sampling strategies [46], may have introduced biases into the study and thereby limited the generalizability of the result. It is possible that subgroups within staff categories (other than those described in the Results section) were not represented in the sample due to the researcher’s unawareness. For example, it cannot be ruled out that a health staff member’s interest in participating in a research study on fall prevention practices may have been affected by their interest in fall prevention. The relatively low number of participants in the study may have contributed further to this risk of sampling bias.

The competence and background of the observing researcher may also have limited the context-specific in-depth understanding of the observations. Although this researcher was knowledgeable in fall prevention due to participating in multidisciplinary research projects, they had no previous training or experience in health care work. Finally, because this study was performed in 1 specific clinical setting, these results may not be generalized to any other in-hospital setting.

### Comparison With Prior Work

The holistic view on in-hospital fall prevention practices expressed by the health staff and observed in the clinical context of this study opens up new possibilities for using fall prevention technologies in hospital settings, such as to support staff activities that prepare patients for long-term fall prevention. This view is well in line with previous research confirming the positive impact of patient and care team partnerships to prevent falls and fall injuries and how this can be supported by human-centered tools [19,20]. The level of importance of contextual support to create more realistic “homelike” conditions during mobility and ADL training, which was pointed out by the staff in this study, is different and thus complements the previously studied technology-based in-hospital fall prevention tools and approaches, which aimed to detect early signs of acutely elevated fall risks and falls, such as those described by Cooper et al [9]. This includes chair sensor alarms [10], video- and image-based monitoring [11,12], wearable devices [13], and artificial intelligence–based motion alarms, such as those mentioned by Pagels [14]. The staff’s request to make the clinical environment more “homelike” can be seen to reflect more holistic models of fall prevention that involve complex interplays between people, activities, processes, organization, environment, and technology, as seen in the models proposed by Choi et al [52] and Taylor and Hignett [15]. Although the review by Hignett and Masud [17] on the associations between inpatient falls and environmental modifications identified a lack of supporting scientific evidence for most recommendations on how to manage environmental hazards in hospital wards, which included flooring, lighting, and bed alarms, it is likely that making the ward’s physical context more “homelike” could introduce new environmental hazards related to falls. As a result, environmental modifications in care settings must be implemented with precaution. Keeping patient rooms intact and introducing homelike elements in other locations outside the rooms is one strategy. Temporarily anchoring mobile, homelike modules in patient rooms offers a more flexible approach, allowing the hospital environment to be tailored to each patient’s training needs. These modules could also incorporate embedded digital systems to support and enhance training. One idea would be to develop modules that could be used both in clinical settings and in-home settings, thus allowing patients to continue the training that they were introduced to in the hospital during their rehabilitation. This would allow them to gradually achieve a more active life after their hospital discharge. Information gained from previous research on how to create meaningful and feasible interactions between older adults and home-based digital systems for fall prevention training, such as that from Ogonowski et al [22], for example, can be of value in the design of these modules. It is also essential to build on the knowledge of how to engage in a creative dialogue with patients regarding their personal goals because incorporating these goals into nonstigmatizing designs of solutions to prevent falls is essential, as described by Bianco et al [21]. The dialogue between staff and patients is also essential when the patients are preparing themselves for the challenges posed by their home settings after hospital discharge [53]. For example, opportunities to experience homelike situations under the supervision of the clinical staff can contribute to strengthening the patients’ experience with their postinjury bodies and thereby contribute to building self-confidence and self-efficacy in gross motor skills.

### Conclusions

The following conclusions can be drawn from this study:

- Expanding the current view of technology-based in-hospital fall prevention to both include computer-based information- and communication systems and digital fall-protection tools aligns well with clinical practice. These technologies will provide support to the health personnel’s activities to engage patients in fall prevention.
- Health professionals see a need for further support in their activities so that they can prepare patients who have been hospitalized for the challenges they will face after their hospital discharge. They also suggested that the clinical environment should contain more home-based elements. These could provide the patients with opportunities to experience homelike situations under the supervision of clinical staff and may contribute to strengthening the patients’ experience with their postinjury bodies.
- The PACT model can serve as a useful framework for supporting observations, and workshops can be fruitful for enabling health personnel to discuss clinical practices for fall prevention and express their need for further support.

## Appendix

Multimedia Appendix 1 Observation protocol and post-observation interview guide.

Multimedia Appendix 2 Workshop paper material.

Multimedia Appendix 3 Main categories and subcategories describing the people involved in fall prevention.

Multimedia Appendix 4 Type of tools used in activities to prevent falls.

Multimedia Appendix 5 Main categories and subcategories describing the context for fall prevention.

## Abbreviations

ADL: activity in daily life
COREQ: Consolidated Criteria for Reporting Qualitative Research
HCD: human-centered design
PACT: people, activities, contexts, and technologies
