# Supplementary material for: Expanding the Design Space for Fall Prevention in Acute Orthopedic Hospital Care: Human-Centered Design Study
Source: JMIR Hum Factors. 2025 Oct 2;12:e73110. doi: 10.2196/73110 (PMC12531586; doi:10.2196/73110)
Supplement: Multimedia Appendix 1 [file humanfactors_v12i1e73110_app1.docx]

Multimedia Appendix 1 Observation protocol and post-observation interview guide (page 1/2)

**Observation protocol**

| **Date** |  |
| --- | --- |
| **The participant’s staff category** |  |
| **Department** |  |

| **Observed aspect** | **The researcher’s observation notes** | **Potential feedback from the participant on the researcher’s observation notes** |
| --- | --- | --- |
| **Context**  ***Physical****(E.g., light, sound, furniture, equipment, free spaces, temperature. Supplemented by a photo of the empty room).*  ***Social****(E.g., if the participant is supported by colleagues or written instructions, if the participant needs to take special consideration for privacy or other social aspects).*  ***Organizational****(E.g., if information transfer to or from the participant occurs in connection with observed events).* |  |  |
| **People in the room**  *E.g., which people are in the room and where they are located, what kind of patient needs (physical conditions, cognitive/psychological, etc.) the participants need to consider during events.”* |  |  |
| **Events/activities**  *E.g., What is happening? What is the target/aim of events/activities? Who are involved? What are the results of events/activities?* |  |  |

| **The researchers own reflection over his/her impressions** |  |
| --- | --- |

Multimedia Appendix 1 Observation protocol and post-observation interview guide (page 2/2)

**Post-observation interview guide**

*Tell me a bit about your work during the shift I observed related to fall prevention work.*

- Do you think that anything unusual happened in your work during the shift?
- Was there anything you did more/less than usual?
- Did you use any specific templates/forms to give/receive reporting before, during, or after the shift? If yes, which ones?
- * Now, I would like us to go through what I have noted about the environment and activities in your work, so you have the opportunity to correct if I have misunderstood something and complement where needed: … [here, the researcher reads points from the protocol and writes down any comments in the protocol].^[[1]](#footnote-1)^

*Is there anything else you want to add that we haven’t talked about?*

1. This question was mostly not used in the interviews since the researcher had had the opportunity to ask questions during the observations. [↑](#footnote-ref-1)
