# Supplementary material for: Expanding the Design Space for Fall Prevention in Acute Orthopedic Hospital Care: Human-Centered Design Study
Source: JMIR Hum Factors. 2025 Oct 2;12:e73110. doi: 10.2196/73110 (PMC12531586; doi:10.2196/73110)

Multimedia Appendix 2 Workshop paper material (page 1/2)

Page 1


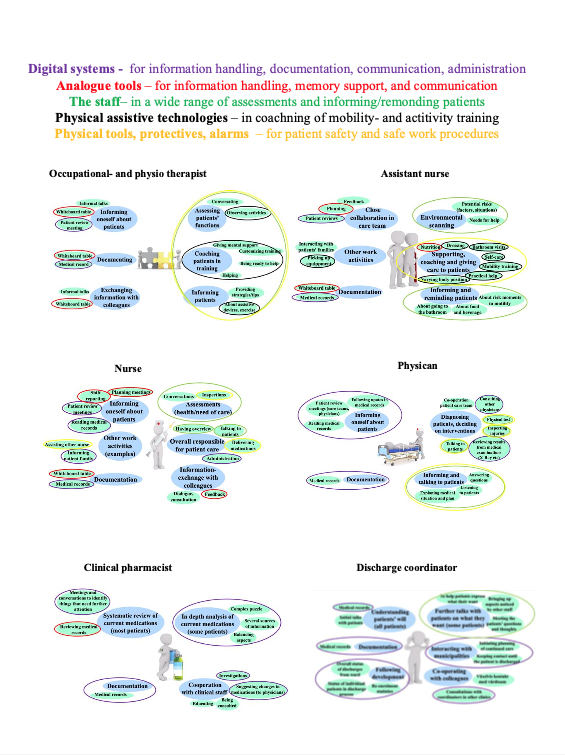


Multimedia Appendix 2 Workshop paper material (page 2/2)

Page 2


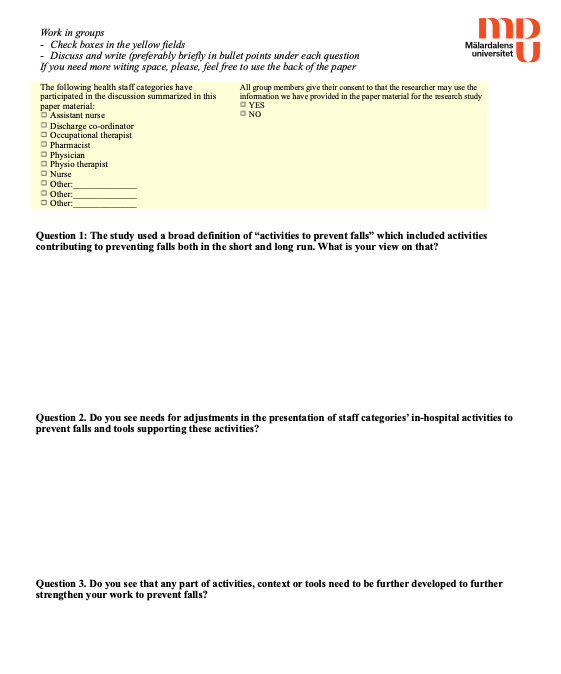

Supplement: Multimedia Appendix 2 [file humanfactors_v12i1e73110_app2.docx]
