# Supplementary material for: Expanding the Design Space for Fall Prevention in Acute Orthopedic Hospital Care: Human-Centered Design Study
Source: JMIR Hum Factors. 2025 Oct 2;12:e73110. doi: 10.2196/73110 (PMC12531586; doi:10.2196/73110)
Supplement: Multimedia Appendix 3 [file humanfactors_v12i1e73110_app3.docx]

Multimedia Appendix 3 Main- and subcategories describing the people involved in fall prevention (page 1/1)

Main- and subcategories describing the people involved in fall prevention.

|  | Characteristics affecting involvement in fall prevention | Examples |
| --- | --- | --- |
|  |  |  |
| **Health personnel** |  |  |
|  | Health occupation license | See “Activities” |
|  | Amount of clinical experience | Health education student |
|  | Specific appointments | Management/coordination |
|  | Individual competences | Language skills |
| **Patients** |  |  |
|  | Overall health status | Reason for seeking acute orthopedic care |
|  |  | Other health problems |
|  | Personal factors | Communication |
|  |  | Self-awareness |
|  |  | Preferences |
|  |  | Life situation (incl. age) |
| **External people** |  |  |
|  | Professional role (staff) | Patient transportation |
|  | Relation to patient (family) | Spouse, child |
