# Supplementary material for: Expanding the Design Space for Fall Prevention in Acute Orthopedic Hospital Care: Human-Centered Design Study
Source: JMIR Hum Factors. 2025 Oct 2;12:e73110. doi: 10.2196/73110 (PMC12531586; doi:10.2196/73110)
Supplement: Multimedia Appendix 4 [file humanfactors_v12i1e73110_app4.docx]

Multimedia Appendix 4 Type of tools used in activities to prevent falls (page 1/1)

|  | Examples of tool(s) | Activities supported | Color in Figures 1-6 |
| --- | --- | --- | --- |
|  |  |  |  |
| **Computer-based systems** |  |  |  |
|  | software for patient medical records | documentation, communication | Purple |
| **Physical tools and systems** |  |  |  |
|  | paper, whiteboard table | memory support, communication | Red |
| **The staff** |  |  |  |
|  | sensory skills (sight), body parts (arms) | assessments, informing patients | Green |
|  |  |  |  |
| **Mechanical aids and assistive devices** |  |  |  |
|  | walkers, elevated toilet seat | coaching mobility- and ADL training | Black |
| **Physical barriers and mechanical tools** |  |  |  |
|  | aprons, sliding mats | close care, lifting patients | Yellow |
