# Supplementary material for: Expanding the Design Space for Fall Prevention in Acute Orthopedic Hospital Care: Human-Centered Design Study
Source: JMIR Hum Factors. 2025 Oct 2;12:e73110. doi: 10.2196/73110 (PMC12531586; doi:10.2196/73110)
Supplement: Multimedia Appendix 5 [file humanfactors_v12i1e73110_app5.docx]

Multimedia Appendix 5 Main- and subcategories describing the context for fall prevention (page 1/2)

|  | Main categories | Sub-categories |
| --- | --- | --- |
|  |  |  |
| **Physical environment** |  |  |
|  | Staff expeditions |  |
|  |  | Meeting tables and office desks |
|  |  | Stationary computers |
|  |  | Care teams’ Whiteboard tables |
|  |  | Rather crowded |
|  | Corridor |  |
|  |  | Light |
|  |  | Mostly silent and calm |
|  |  | Personnel walking, talking |
|  |  | Patient families waiting |
|  | Patient rooms |  |
|  |  | Single, hallway-bedroom-bathroom |
|  |  | Door slightly open to corridor |
|  |  | Spacious, empty floor areas |
|  |  | Light and well-illuminated |
|  |  | Sterile and clean |
|  |  | Alarm buttons |
|  |  | Wall-mounted furniture |
|  |  | Height-adjustable bed, safety rails |
|  |  |  |
| **Social context** |  |  |
|  | Health personnel-patient | Goal-oriented |
|  |  | Patient-centered and susceptible to needs |
|  |  | Clear and calm |
|  |  | Respecting privacy |
|  |  | Reaching the patients |
|  |  | Meeting patients expressing feelings and trust |
|  |  |  |
|  | Between health personnel |  |
|  |  | Clear roles and hierarchy |
|  |  | Close co-operation |
|  |  | Friendly and positive |
|  |  | Formal and informal meetings |
|  |  | Consultation and support |

Multimedia Appendix 5 Main- and subcategories describing the context for fall prevention (page 2/2)

| **Organizational context** |  |  |
| --- | --- | --- |
|  | Formal operational framework | Demarcations (mission, care capacity) |
|  |  | Regulations, steering documents |
|  |  |  |
|  | Clear and formal work structure |  |
|  |  | Staff meetings and consultations |
|  |  | Patient review |
|  |  | Documentation |
|  |  |  |
|  | Formal roles and hierarchy in decisions and responsibilities | In patient medical care and care |
|  |  | In consultation, collegial support |
|  |  |  |
|  | Structured care process | Enrollment |
|  |  | Diagnosis |
|  |  | Treatment and care |
|  |  | Ready for discharge/discharge |
|  |  |  |
|  | Varied workload | Irregular work hours |
|  |  | Sudden interruptions |
|  |  | Cognitive and mental load |
|  |  | Collegial support |
|  |  |  |
|  | Administrative systems | Documentation (medical records) |
|  |  | Registering patients (to surgery etc.) |
|  |  | Prescribed medications |
|  |  | Medical images |
